# Sudden death due to paralysis and synaptic and behavioral deficits when *Hip14/Zdhhc17* is deleted in adult mice

**DOI:** 10.1186/s12915-016-0333-7

**Published:** 2016-12-07

**Authors:** Shaun S. Sanders, Matthew P. Parsons, Katherine K. N. Mui, Amber L. Southwell, Sonia Franciosi, Daphne Cheung, Sabine Waltl, Lynn A. Raymond, Michael R. Hayden

**Affiliations:** 1 Centre for Molecular Medicine and Therapeutics, Department of Medical Genetics, Child & Family Research Institute, University of British Columbia (UBC), Vancouver, BC V5Z 4H4 Canada; 2 Department of Psychiatry, Brain Research Centre and Djavad Mowafaghian Centre for Brain Health, UBC, Vancouver, BC V6T 1Z3 Canada; 3 Present address: Division of Biomedical Sciences, Faculty of Medicine, Memorial University, Newfoundland and Labrador, A1B 3V6 Canada

**Keywords:** Huntington’s disease, Palmitoylation, Palmitoyl acyltransferase, HIP14, DHHC17

## Abstract

**Background:**

Palmitoylation, the addition of palmitate to proteins by palmitoyl acyltransferases (PATs), is an important regulator of synaptic protein localization and function. Many palmitoylated proteins and PATs have been implicated in neuropsychiatric diseases, including Huntington disease, schizophrenia, amyotrophic lateral sclerosis, Alzheimer disease, and X-linked intellectual disability. HIP14/DHHC17 is the most conserved PAT that palmitoylates many synaptic proteins. *Hip14* hypomorphic mice have behavioral and synaptic deficits. However, the phenotype is developmental; thus, a model of post-developmental loss of *Hip14* was generated to examine the role of HIP14 in synaptic function in the adult.

**Results:**

Ten weeks after *Hip14* deletion (*iHip14* ^*Δ/Δ*^), mice die suddenly from rapidly progressive paralysis*.* Prior to death the mice exhibit motor deficits, increased escape response during tests of anxiety, anhedonia, a symptom indicative of depressive-like behavior, and striatal synaptic deficits, including reduced probability of transmitter release and increased amplitude but decreased frequency of spontaneous post-synaptic currents. The mice also have increased brain weight due to microgliosis and astrogliosis in the cortex.

**Conclusions:**

Behavioral changes and electrophysiological measures suggest striatal dysfunction in *iHip14* ^*Δ/Δ*^ mice, and increased cortical volume due to astrogliosis and microgliosis suggests a novel role for HIP14 in glia. These data suggest that HIP14 is essential for maintenance of life and neuronal integrity in the adult mouse.

**Electronic supplementary material:**

The online version of this article (doi:10.1186/s12915-016-0333-7) contains supplementary material, which is available to authorized users.

## Background

In recent years palmitoylation has emerged as an important regulator of protein localization and function, particularly in neurons [1, 2]. Palmitoylation is the reversible addition of long chain fatty acids, typically palmitate, to proteins at cysteine residues [3, 4]. It is mediated by DHHC-domain containing palmitoyl acyltransferases (PATs) that palmitoylate proteins at cysteine residues via a thioester bond [5, 6]. Many PATs have been implicated in diseases of the nervous system, including Huntington disease (HD), an autosomal dominant fatal neurodegenerative disease; schizophrenia; amyotrophic lateral sclerosis; Alzheimer disease; and X-linked intellectual disability [1, 2].

Palmitoylation is the only reversible lipid modification, and this reversibility is analogous to phosphorylation, where enzyme-mediated addition and removal of palmitate allows for rapid cycling of palmitate on some proteins, providing an additional level of regulation of localization and function [7]. Indeed, in neurons, palmitoylation has been shown to regulate localization of many synaptic proteins. For example, palmitoylation of post-synaptic density protein 95 (PSD95) is required for its synaptic localization, and its palmitoylation undergoes cycles of de/repalmitoylation that regulate PSD95 nanoclusters within the synapse [8]. Palmitoylation also regulates the synaptic insertion/removal of α-amino-3-hydroxy-5-methyl-4-isoxazolepropionic acid receptor (AMPAR) subunits GluA1 and GluA2, and of *N*-methyl-d-aspartate receptor (NMDAR) subunits GluN2A and GluN2B [9, 10].

Huntingtin interacting protein 14 (HIP14 or ZDHHC17) is the most highly conserved of the 23 human PATs. It palmitoylates many synaptic proteins, including cysteine string protein (CSP), GluA1, GluA2, PSD95, synaptosomal-associated protein 25 (SNAP25), synaptotagmin 1 (SYT1), the large conductance calcium- and voltage-activated potassium BK channel (KCNMA1) STREX isoform, and the HD disease-causing protein huntingtin (HTT) [2]. It has recently become more apparent that HIP14 is an important regulator of synaptic function. Indeed, *Hip14* knockdown reduces PSD95 clustering in neurons [6] and in *Drosophila melanogaster* HIP14 is required for CSP targeting to synaptic vesicles and, in turn, pre-synaptic exocytosis [11]. Interestingly, in an HD mouse model HIP14 is less active [12, 13] and the constitutive *Hip14*-deficient mouse (*Hip14* ^*gt/gt*^) has behavioral, neuropathological, and synaptic dysfunction reminiscent of HD [12, 14, 15].

The *Hip14* ^*gt/gt*^ mouse is a hypomorph expressing ~10% of endogenous HIP14 protein [16, 17] and the phenotype is developmental, as neurodegeneration occurs during late embryogenesis. Thus, we sought to determine the consequences of complete loss of *Hip14* in the adult animal and its effect on synaptic deficits and neuronal degeneration. An inducible *Hip14*-deficient mouse model was generated, and *Hip14* deletion was induced in the young adult mouse.

## Results

### Generation of post-development *Hip14*-deficient mice

**Fig. 1:**
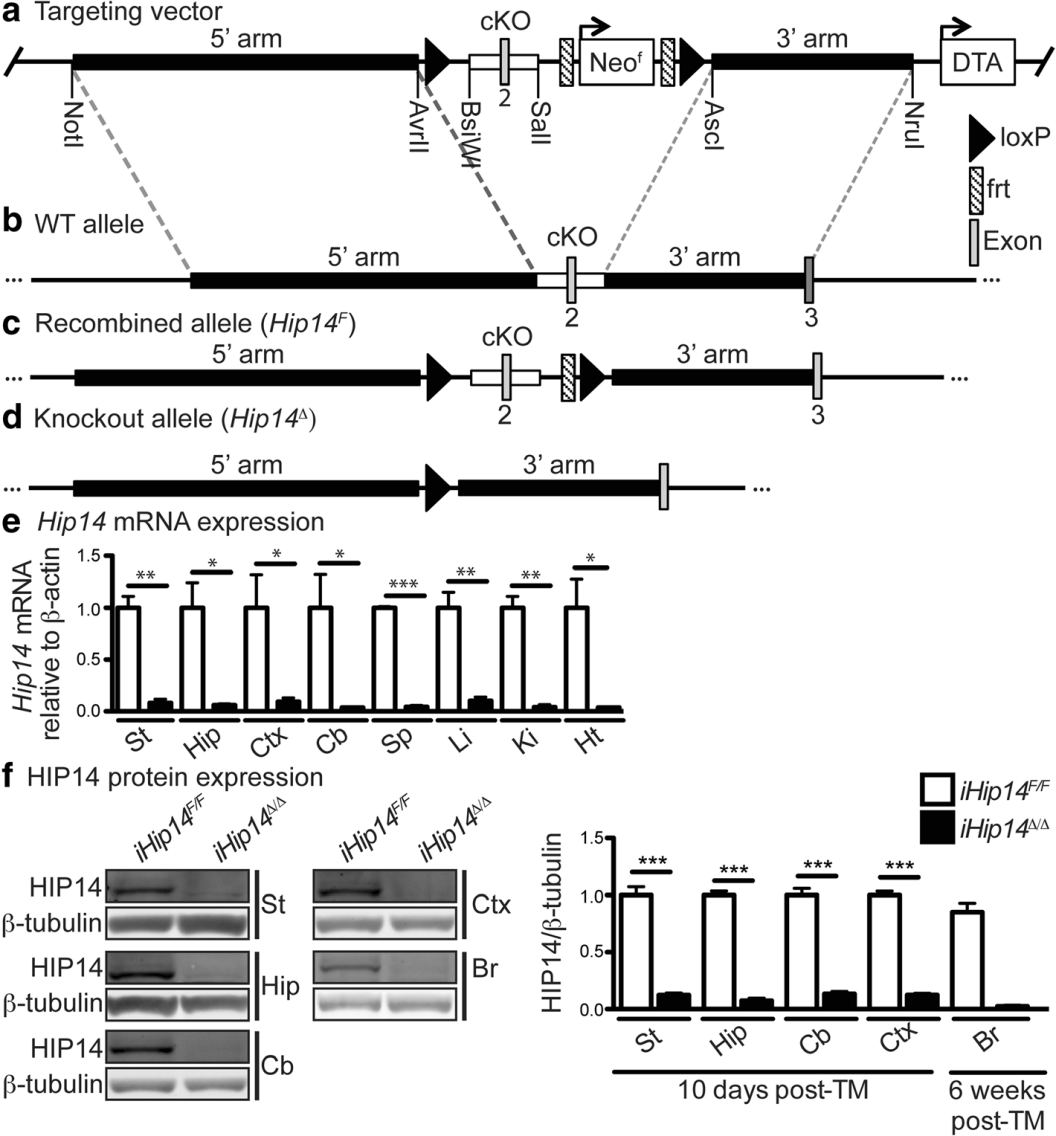
Generation of *Hip14* conditional knockout mice. The targeting vector that was used is shown in (**a**). It was generated using PCR cloning of the 5′ and 3′ homology arms (5.5 and 3.2 kbp, respectively) and the deletion region (conditional knockout region [*cKO*]) with the indicated restriction enzyme sites added by PCR and used for cloning into the targeting vector, such that loxP sites are oriented in the same direction up and downstream of the cKO region. The deletion region, in *gray*, includes exon 2 and upstream and downstream intronic sequences to a total of 1.1 kbp. The targeting vector also includes a positive neomycin (*Neo*) selection cassette flanked by flippase recognition target (*frt*) sites and a negative diphtheria toxin A (*DTA*) selection cassette outside of the homology arms. The wild-type (*WT*) allele is shown in (**b**) with the 5′ and 3′ homology arms and the cKO region indicated. The recombined allele (*Hip14* ^*F*^) is shown in (**c**). The neo cassette was removed during embryonic cell culture after targeting by electroporation with Flp recombinase and negative selection with G418. The knockout allele following expression of Cre recombinase is shown in (**d**) where recombination between the loxP sites occurs by the action of Cre leading to deletion of the cKO region, including exon 2. This deletion results in a frameshift mutation and multiple premature stop codons. The expression of *Hip14* mRNA in the striatum, hippocampus, cortex, cerebellum, spleen, liver, kidney, and heart in *iHip14* ^*Δ/Δ*^ mice and in *iHip14* ^*F/F*^ control mice relative to β-actin is shown in (**e**) (*N* = 3). There was a 90% or greater decrease in *Hip14* mRNA in *iHip14* ^*Δ/Δ*^ mice compared to control mice. **f** HIP14 protein expression in the striatum, hippocampus, cerebellum, and cortex from *iHip14* ^*Δ/Δ*^ and *iHip14* ^*F/F*^ mice, where the tissues were collected 10 days post-TM treatment, is shown. Also shown is whole brain collected 6 weeks post-TM treatment from *iHip14* ^*Δ/Δ*^ and *iHip14* ^*F/F*^ mice. Whole cell lysate was run on western blot and probed with anti-HIP14 and anti-β-tubulin antibodies. The HIP14 immunoblot is the *top panel* in each set of images; the β-tubulin is in the *bottom panel*. The amount of HIP14 protein expressed is quantified in the graph relative to β-tubulin expression (*N* = 2–10). A 90% or greater decrease in HIP14 was observed in *iHip14* ^*Δ/Δ*^ mice. Data were analyzed using Student’s *t* test

*Hip14* “conditional knockout” (*Hip14* ^*F/F*^) mice (Fig. 1a–d) were crossed to ubiquitously expressed tamoxifen (TM)-inducible Cre recombinase (Cre-ER^T2^)-expressing transgenic mice [18]. *Hip14* deletion was induced in *Hip14* ^*F/F*^;Cre + mice at 6 weeks of age by TM treatment (*iHip14* ^*Δ/Δ*^ herein) to allow mice a month to recover from TM toxicity prior to any behavior testing performed at 3 months of age [19].

*Hip14* mRNA and protein levels were assessed at 10 days after the last injection and 6 weeks post-induction to assess deletion efficiency compared to *Hip14* ^*F/F*^;Cre– TM control mice (*iHip14* ^*F/F*^ herein). *Hip14* mRNA and protein expression was decreased by >90% 10 days post-TM treatment in all brain regions and peripheral tissues tested (Fig. 1e and f). Greater than 95% loss of HIP14 protein was observed in the whole brain at 6 weeks post-TM treatment (Fig. 1f). These data indicate that deletion of *Hip14* in *iHip14* ^*Δ/Δ*^ mice is >90% effective.

### Low body weight and hyperactivity in *iHip14*^*Δ/Δ*^ mice

**Fig. 2:**
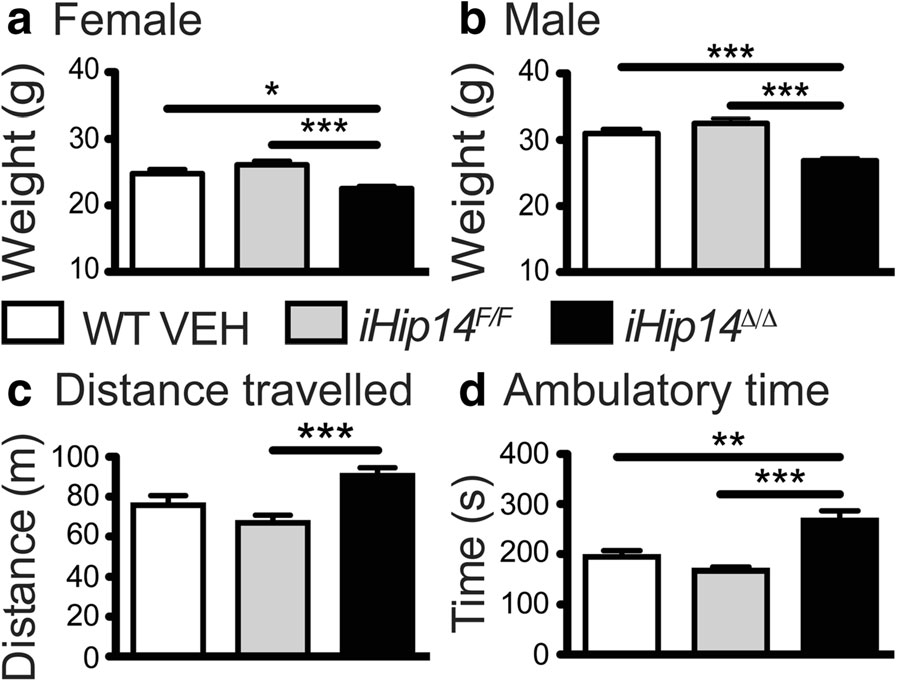
Decreased body weight and hyperactivity in 3-month old *iHip14* ^*Δ/Δ*^ mice. A ~10% decrease in body weight of female (**a**; analysis of variance, *ANOVA*: *p* < 0.0001) and male (**b**; ANOVA: *p* < 0.0001) *iHip14* ^*Δ/Δ*^ mice compared to WT VEH and *iHip14* ^*F/F*^ mice was observed (*N* = 16–24). Spontaneous activity was assessed by infrared beam breaks during a 30-min exploration of a 27 × 27 × 20.3 cm box at 3 months of age. Total distance traveled (**c**) and ambulatory time as assessed by consecutive beam breaks (**d**) were assessed. On both measures the *iHip14* ^*Δ/Δ*^ mice were hyperactive compared to controls (distance traveled ANOVA: *p =* 0.0005; ambulatory time ANOVA: *p* = <0.0001; ambulatory episodes ANOVA *p* = 0.0001; *N* = 13–18)

To assess overall health, *iHip14* ^*Δ/Δ*^ mice were weighed at 3 months of age, approximately 7 weeks post-induction. Both female and male *iHip14* ^*Δ/Δ*^ mice were approximately 10% smaller than wild-type (WT) vehicle (VEH)-treated and *iHip14* ^*F/F*^ control mice (Fig. 2a and b). To assess global nervous system and motor function, spontaneous activity was assessed during the dark phase. *iHip14* ^*Δ/Δ*^ mice were hyperactive during exploration of a novel environment (increased distance traveled, Fig. 2c, and ambulatory time, d).

### Motor coordination and sensorimotor gating deficits in *iHip14*^*Δ/Δ*^ mice

**Fig. 3:**
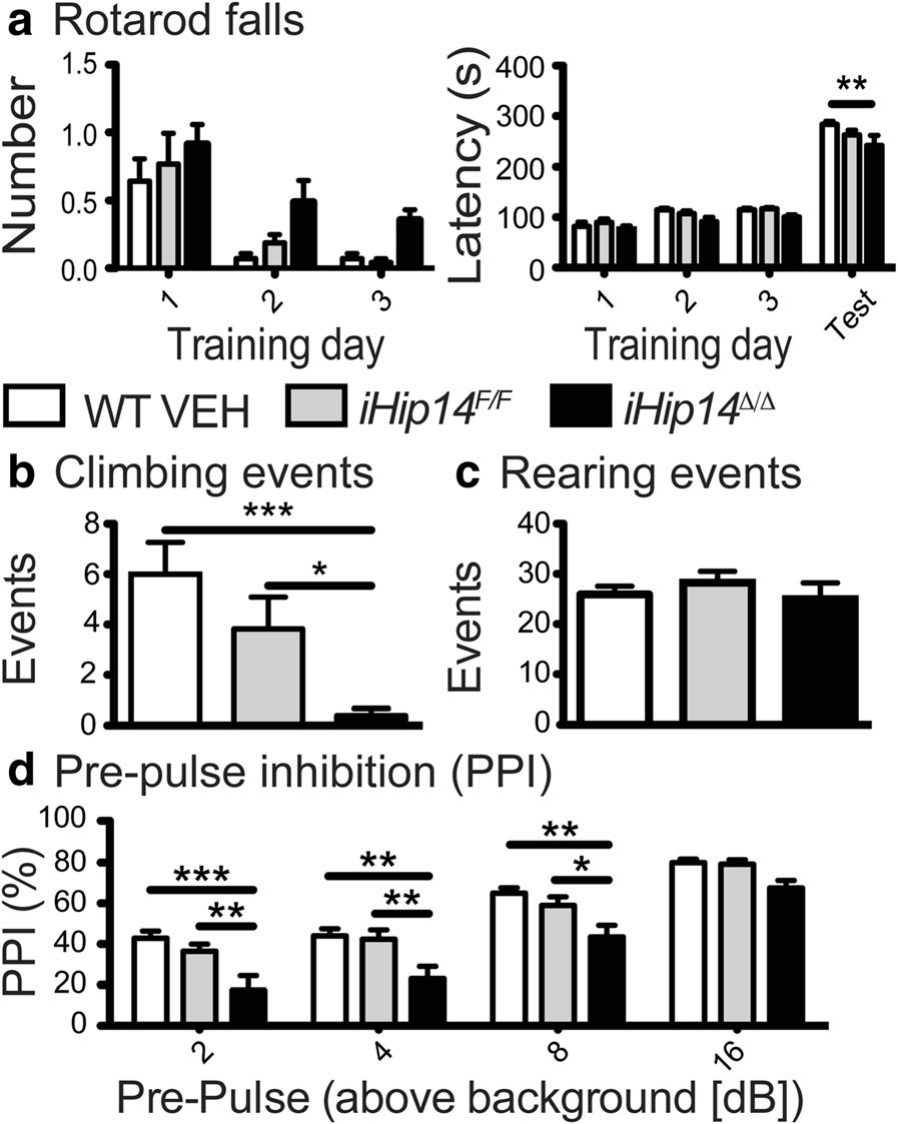
Motor coordination and sensorimotor gating deficits in 3-month old *iHip14* ^*Δ/Δ*^ mice. Over three consecutive days, 3-month-old mice were trained on a fixed-speed rotarod and tested on an accelerating rotarod on the fourth day (**a**). The number of falls (*left*) and the latency to the first fall (*right*) were recorded. *iHip14* ^*Δ/Δ*^ mice fell off the rotarod more (two-way ANOVA: genotype *p* = 0.0053, *p* < 0.0001; interaction *p* = 0.9071; *N* = 14–16) and sooner (two-way ANOVA: genotype *p* = 0.0009, training day *p* < 0.0001, interaction *p* = 0.4581; *N* = 14–16) both during training and during testing. Mice were allowed to freely explore and climb a wire mesh container. *iHip14* ^*Δ/Δ*^ mice climbed fewer times (**b**; ANOVA: *p* = 0.001; *N* = 13–18) but did not rear less (**c**; ANOVA: *p* = 0.65; *N* = 13–18) compared to control mice. Pre-pulse inhibition (*PPI*) was measured as the percentage of decrease in the magnitude (velocity) of startle with a pre-pulse compared to the magnitude of startle without a pre-pulse. *iHip14* ^*Δ/Δ*^ mice had a PPI deficit compared to controls (**d**; repeated measures ANOVA: genotype *p* < 0.0001, pre-pulse *p* < 0.0001; *N* = 15–18)

To determine if loss of HIP14 in the adult mouse results in neurological dysfunction, motor function was assessed. Motor coordination of *iHip14* ^*Δ/Δ*^ mice was tested on rotarod and climbing tests [20]. *iHip14* ^*Δ/Δ*^ mice had motor coordination deficits on the rotarod compared to control WT VEH and *iHip14* ^*F/F*^ mice (Fig. 3a). As the rotarod performance is a trained test where mice learn to stay on the rotarod, it is less sensitive to motor dysfunction than the spontaneous test of motor coordination: climbing [21, 22]. There was also a dramatic reduction in the number of climbing events in these mice (Fig. 3b) but no change in the number of rearing events, indicative of motivation to explore the apparatus (Fig. 3c). Taken together, these data indicate motor dysfunction.

Schizophrenia and other neurological disorders were recently shown to be enriched for palmitoylated proteins [1]. Pre-pulse inhibition (PPI) is a test of sensorimotor gating, partly mediated by the striatum [20, 23]. PPI deficits are associated with schizophrenia and other psychiatric disorders as well as HD [24]. When a quieter tone (the pre-pulse) is played prior to a loud stimulus (the startle pulse), mice with intact sensorimotor gating will startle less than they would to the loud startle stimulus alone [25]. *iHip14* ^*Δ/Δ*^ mice showed impaired pre-pulse inhibition at all pre-pulse levels that was significant at 2, 4, and 9 dB above background with a trend at 16 dB compared to control mice (Fig. 3d), indicating impaired sensorimotor gating and potential striatal dysfunction.

### Increased escape response and anhedonia in *iHip14*^*Δ/Δ*^ mice

**Fig. 4:**
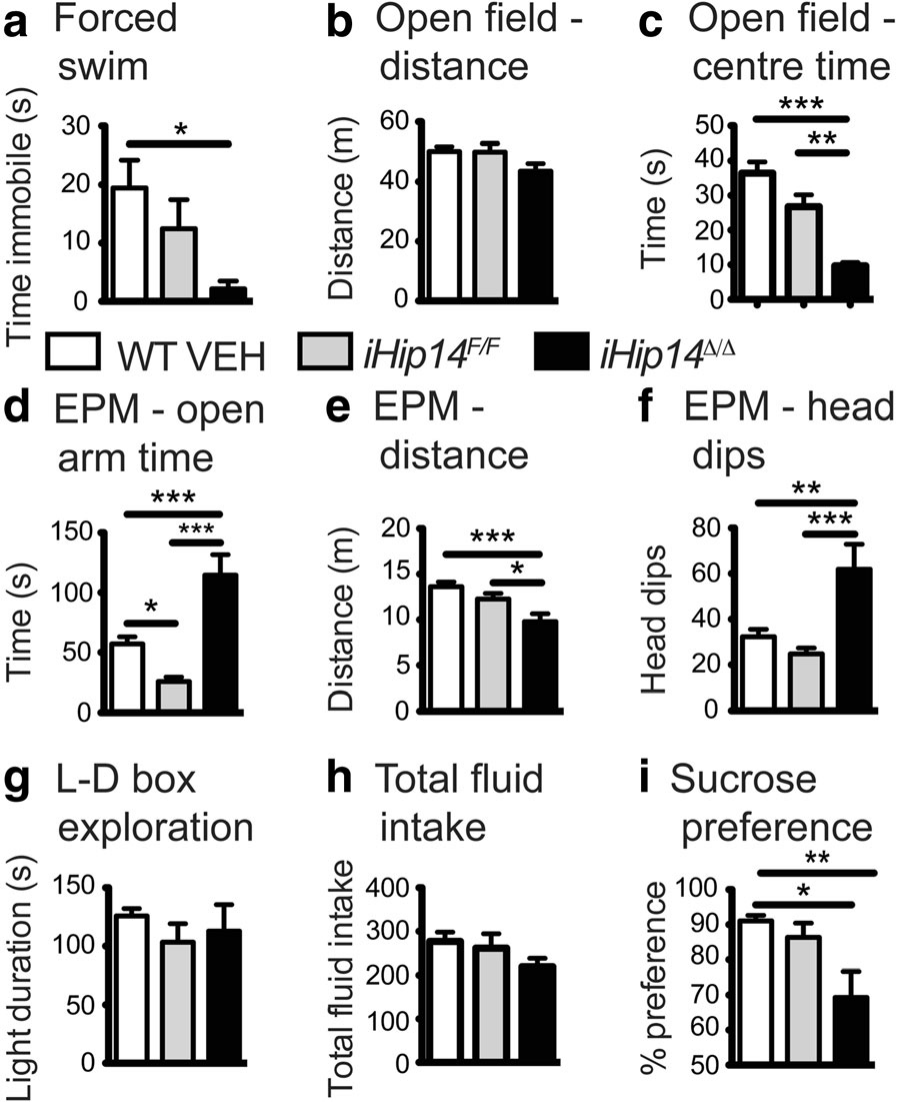
Three-month-old *iHip14* ^*Δ/Δ*^ mice have increased escape response and display anhedonic-like behavior. *iHip14* ^*Δ/Δ*^ mice spent significantly less time immobile in the forced swim test (**a**; ANOVA: *p* = 0.0272; *N* = 15–19). Mice were placed in an open field under bright lighting. *iHip14* ^*Δ/Δ*^ mice explored the field to the same extent as the control mice (**b**; ANOVA *p* = 0.0844; *N* = 24–25) but spent less time in the center (**c**; ANOVA: *p* < 0.0001; *N* = 24–25). *iHip14* ^*Δ/Δ*^ mice spent more time in the open arms of the elevated plus maze (**d**; ANOVA: *p* < 0.0001; *N* = 25–34; *EPM*), explored the maze less (**e**; ANOVA: *p* = 0.0007; *N* = 25–34), and dipped their heads off the edge of the open arms more (**f**; ANOVA: *p* = 0.0001; *N* = 25–34) than controls. Mice were placed in an enclosed box with a brightly lit side and a dark side. *iHip14* ^*Δ/Δ*^ mice spent the same amount of the time in the light box as control mice (**g**; ANOVA: *p* = 0.58; *N* = 13–16)*.* Mice were allowed free access to a 2% sucrose solution and water over a 24-h period, and the total fluid consumption (g/kg of body weight; **h**) was measured. *iHip14* ^*Δ/Δ*^ mice had no change in total fluid intake (**h**; ANOVA: *p* = 0.27; *N* = 14–16) but had decreased preference for the sucrose solution (**i**; ANOVA: *p* = 0.0066; *N* = 14–16)

As it is becoming increasingly evident that palmitoylation is important in neuropsychiatric disorders [1, 2], the impact of loss of HIP14 on psychiatric phenotypes such as depression and anxiety was assessed [21, 26]. *iHip14* ^*Δ/Δ*^ mice were tested in the Porsolt forced swim test for depression [26–28]. Interestingly, *iHip14* ^*Δ/Δ*^ mice spent dramatically less time immobile during forced swimming than controls (Fig. 4a). During behavior testing *iHip14* ^*Δ/Δ*^ mice were observed to be very reactive to the experimenter and testing conditions, having explosive responses to both. Thus, rather than truly reflecting an anti-depressive effect, these data in the forced swim test are consistent with the hyperactivity and reactivity to testing observed in these mice.

Anxiety-like behavior was assessed in the open field exploration test, a well-established test of anxiety-like behaviors in rodents [21, 29]. *iHip14* ^*Δ/Δ*^ mice explored the brightly lit open field to the same extent as control mice, as measured by distance traveled (Fig. 4b), but spent less time in the center of the field (Fig. 4c), suggesting an increase in anxiety-like behaviors in these mice.

To confirm anxiety in *iHip14* ^*Δ/Δ*^ mice, the mice were tested using the elevated plus maze (EPM) test for anxiety [30, 31]. Surprisingly, the *iHip14* ^*Δ/Δ*^ mice spent more time in the open arms of the EPM than the control WT VEH or *iHip14* ^*F/F*^ mice (Fig. 4d), suggesting decreased anxiety, opposite to the findings from the open field testing. The *iHip14* ^*Δ/Δ*^ mice did not explore the EPM as much as the control mice (Fig. 4e), likely because they spent more time dipping their head off the edge of the open arms of the maze (Fig. 4f), again suggesting decreased anxiety. These data suggest an anxiolytic phenotype rather than the anxiogenic phenotype suggested by the open field exploration test. Alternatively, the *iHip14* ^*Δ/Δ*^ mice may be trying to escape the testing apparatus; i.e., they spend more time exploring the edges of the open field box trying to find a way out, and in the EPM they dip their heads off the open arms trying to escape the maze. This interpretation would also be consistent with their reactivity to handling and testing and reduced time spent immobile in the forced swim test.

To separate anxiety-like behavior from increased escape response, a modified light-dark box test was designed that completely removed any possibility of escape, where one side was dark and the other side was brightly lit, and both sides were completely enclosed. The *iHip14* ^*Δ/Δ*^ mice spent the same amount of time in the light box as the control mice (Fig. 4g). These data suggest *iHip14* ^*Δ/Δ*^ mice are not anxious per se but have an increased escape response.

To delineate escape response from depressive-like behavior, the *iHip14* ^*Δ/Δ*^ mice were tested using the sucrose preference test for anhedonia-like behavior (the inability to experience pleasure), as anhedonia is a major symptom of depression [26, 32]. The sucrose preference test is performed in the home cage with no experimenter present, thus eliminating the confound of increased escape response. The *iHip14* ^*Δ/Δ*^ mice consumed the same amount of fluid (Fig. 4g) but had decreased preference for sucrose compared to the control mice (Fig. 4h), indicating anhedonia and suggesting a depressive-like phenotype.

### Increased forebrain weight, increased cortical volume, and decreased corpus callosum volume in *iHip14*^*Δ/Δ*^ mice

**Fig. 5:**
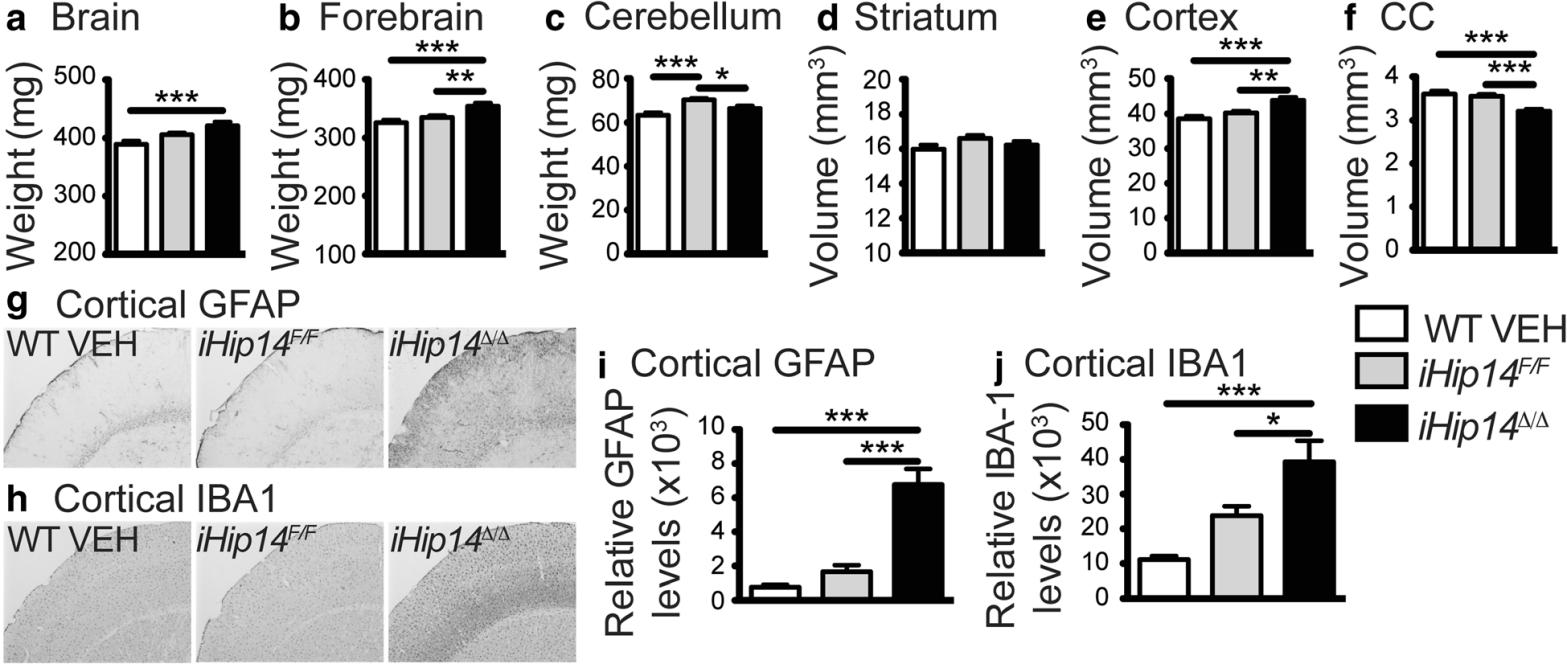
Three-month-old *iHip14* ^*Δ/Δ*^ mice have increased brain and forebrain weight, increased cortical volume, and decreased corpus callosum volume. *iHip14* ^*Δ/Δ*^ mice have increased brain weight compared to control mice (**a**; ANOVA: *p* = 0.0004; *N* = 13–18), larger forebrain weight (**b**; ANOVA: *p* = 0.0002; *N* = 13–18), and unchanged cerebellum weight (**c**; ANOVA: *p* < 0.0001; *N* = 12–18). Brains were then sectioned and stained with NeuN to stain neurons, and striatal (**d**), cortical (**e**), and corpus callosum (**f**; *CC*) volume were determined. No change in striatal volume was observed (**d**; ANOVA: *p* = 0.43; *N* = 18–23) in *iHip14* ^*Δ/Δ*^ mice, but there was a significant increase in cortical volume (**e**; ANOVA: *p* < 0.0001; *N* = 13–18) and decrease in corpus callosum volume (**f**; ANOVA: *p* < 0.0001; *N* = 13–18). Sections were stained with antibodies against glial fibrillary acidic protein (GFAP) or ionized calcium-binding adapter molecule 1 (IBA1) to stain astrocytes (**g** and **i**) and microglia (**h** and **j**). There was increased staining intensity of GFAP (**g** and **i**; ANOVA: *p* < 0.0001; *N* = 7–9) and IBA1 in *iHip14* ^*Δ/Δ*^ cortex (**h** and **j**; ANOVA: *p* = 0.0001; *N* = 7–9)

To determine the effect of loss of HIP14 in the adult mouse on brain morphology and neurodegeneration, neuropathological assessments were performed. Increased brain weight was observed in *iHip14* ^*Δ/Δ*^ mice (Fig. 5a). This increase was restricted to the forebrain (Fig. 5b), as there was no change in cerebellar weight compared to WT VEH control mice (Fig. 5c). Also, unexpectedly, there was no change in striatal volume in the *iHip14* ^*Δ/Δ*^ mice compared to controls (Fig. 5d), but there was an increase in cortical volume (Fig. 5e) and a decreased corpus callosum volume, indicating loss of white matter (Fig. 5f) potentially due to axonal degeneration or loss of myelination.

To understand what factors may account for the observed increase in cortical volume, astrocytes and microglia were assessed by glial fibrillary acidic protein (GFAP) and ionized calcium-binding adapter molecule 1 (IBA1) staining intensity, respectively. There was a dramatic increase in both GFAP (Fig. 5g and i) and IBA1 (Fig. 5h and j) staining intensity in the cortex of *iHip14* ^*Δ/Δ*^ mice compared to controls, indicating significant astrogliosis and microgliosis, respectively.

### Impaired synaptic transmission in the striatum of *iHip14*^*Δ/Δ*^ mice

**Fig. 6:**
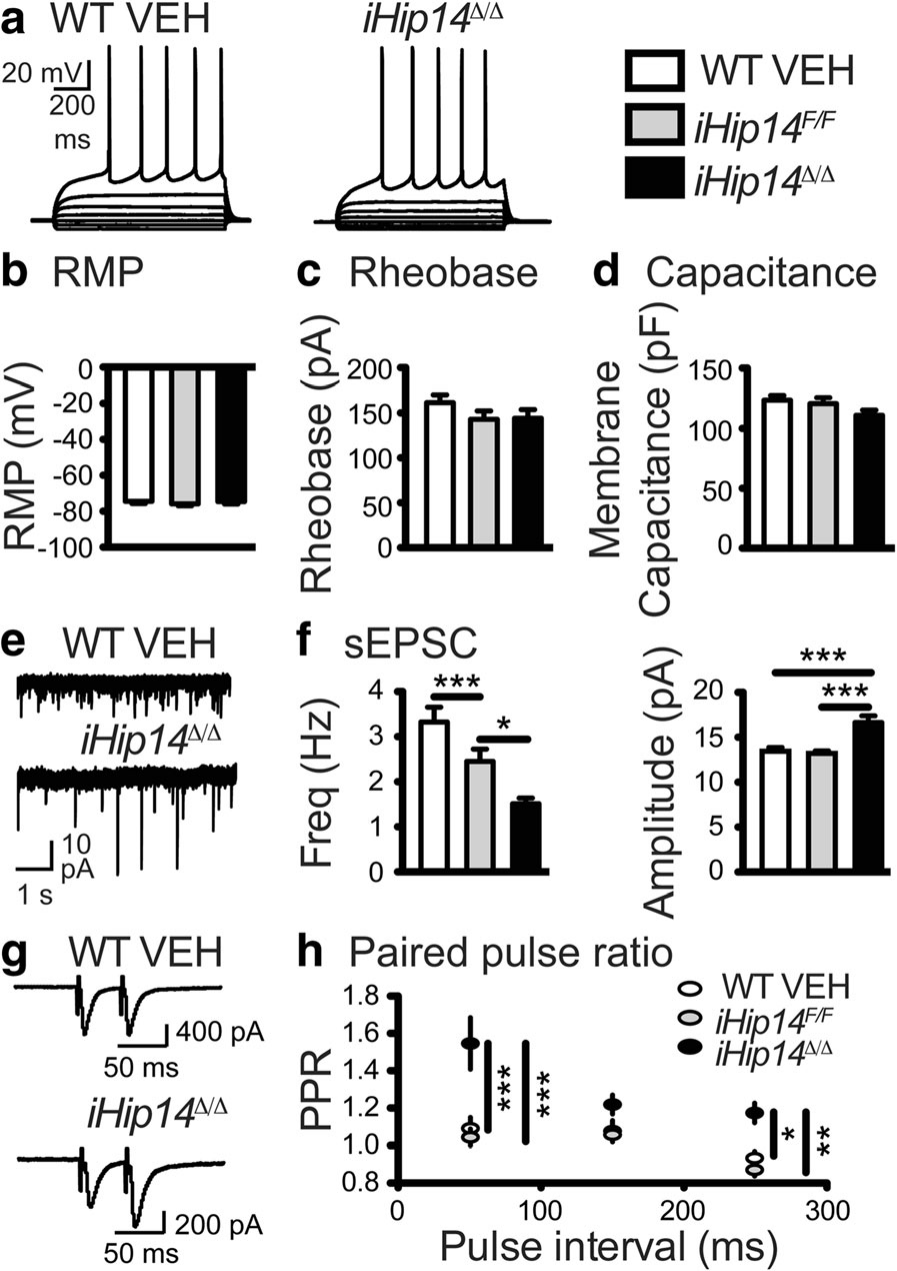
Impaired synaptic transmission in *iHip14* ^*Δ/Δ*^ MSNs.MSNs in the central dorsal striatum were whole-cell patch clamped in acute coronal slices from 3-month-old mice. A representative trace of current-clamp membrane potential responses to a series of current injections (from –100 pA to 200 pA in 50-pA increments) is shown in (**a**). *iHip14* ^*Δ/Δ*^ MSNs had the same resting membrane potential (*RMP*) (**b**: ANOVA: *p* = 0.68; *N* = 21–25), fired at the same rheobase current (**c**; ANOVA: *p* = 0.30; *N* = 21–25), and had the same membrane capacitance (**d**; ANOVA: *p* = 0.094; *N* = 31–43). *iHip14* ^*Δ/Δ*^ mice had decreased frequency (**f** *left*; ANOVA: *p* < 0.0001; *N* = 19–23) but increased amplitude (**f** *right*; ANOVA: *p* < 0.0001; *N* = 19–23) of spontaneous excitatory post-synaptic currents (*sEPSC*), representative traces are shown in (**e**). *iHip14* ^*Δ/Δ*^ mice had increased paired pulse ratios (**h**; *PPR*; two-way ANOVA: genotype *p* = 0.0001; pulse interval *p* < 0.0001; interaction *p* = 0.0047; *N* = 8–10), representative traces are shown in (**g**)

Since palmitoylation has been implicated in localization of synaptic proteins and in synaptic signaling and HIP14 was previously shown to be important for striatal physiology and striatal processing during motor behaviors [14, 15], the synaptic properties of medium-sized spiny neurons (MSNs) in the striatum of *iHip14* ^*Δ/Δ*^ mice were examined [14, 33–35] by making current- and voltage-clamp recordings in the dorsal striatum. We observed no significant effect of loss of *Hip14* on either MSN resting membrane potential or rheobase (Fig. 6a–c): the amount of current injection required to initiate action potential firing. Membrane capacitance, an indirect measure of cell-surface area, was also similar between groups (Fig. 6d). Thus, MSN membrane potential, excitability, and cell size appear to be unaltered by loss of *Hip14* in adulthood.

To assay excitatory synaptic function, AMPAR-mediated spontaneous excitatory post-synaptic currents (sEPSCs) were recorded from MSNs held at –70 mV in the presence of picrotoxin, a γ-aminobutyric acid A (GABA_A_) receptor antagonist. There was a significant decrease in the frequency and a significant increase in the amplitude of sEPSCs recorded from *iHip14* ^*Δ/Δ*^ MSNs (Fig. 6e and f) compared to controls. These data demonstrate synaptic dysfunction in *iHip14* ^*∆/∆*^ mice and suggest a reduction in the number of excitatory synapses and/or a reduction in transmitter release probability with additional AMPARs at the synapses or more glutamate released per synaptic vesicle.

To assess transmitter release probability from cortical afferents onto MSNs in the striatum, a stimulating electrode was placed 200–250 μm dorsal to the recorded cell, various inter-pulse intervals were applied, and the paired pulse ratio (PPR) was calculated. MSNs from *iHip14* ^*Δ/Δ*^ mice had increased PPRs compared to MSNs from control mice (Fig. 6g and h). These data are indicative of a lower probability of transmitter release and are consistent with the reduction in sEPSC frequency, further suggesting synaptic dysfunction.

### Reduced survival due to rapidly progressing paralysis in *iHip14*^*Δ/Δ*^ mice

**Fig. 7:**
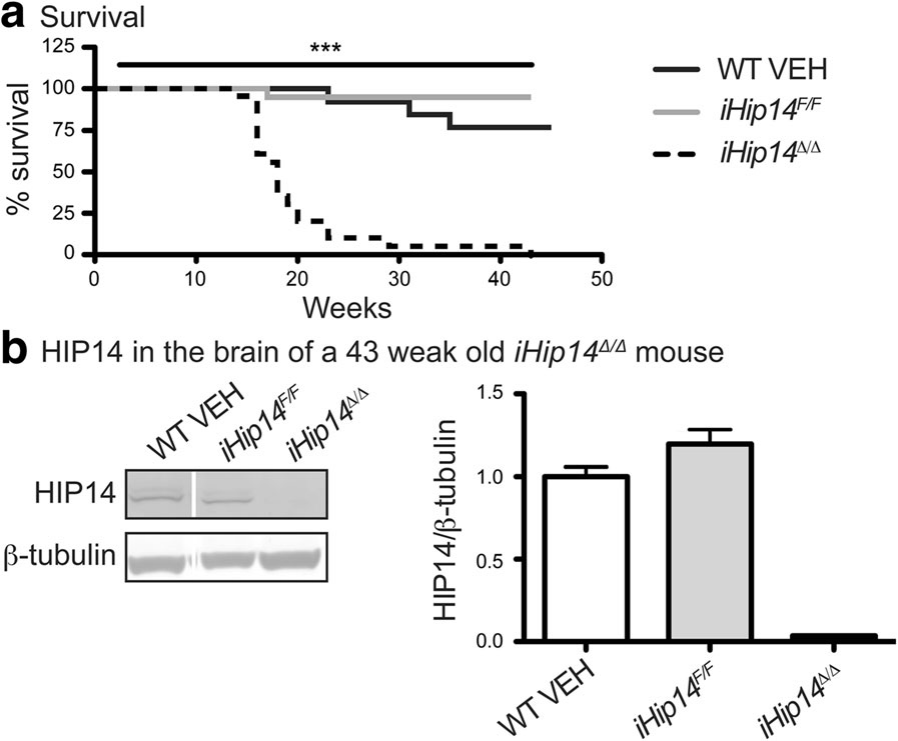
Reduced survival in *iHip14* ^*Δ/Δ*^ mice and HIP14 protein expression in the brain of a 43-week-old *iHip14* ^*Δ/Δ*^ mouse. A dramatic reduction in survival was observed in the *iHip14* ^*Δ/Δ*^ mice compared to controls (**a**; log-rank test: *X* ^2^(4) = 93.76; *N* = 11–24). **b** Whole cell lysate was run on western blot and probed with anti-HIP14 and anti-β-tubulin antibodies. The HIP14 immunoblot is the *top panel* and that of the β-tubulin is in the *bottom panel*. The amount of HIP14 protein expressed is quantified in the graph relative to β-tubulin expression (*N* = 3 WT VEH, 2 *iHip14* ^*F/F*^, and 1 *iHip14* ^*Δ/Δ*^). A 97% decrease in HIP14 was observed in the *iHip14* ^*Δ/Δ*^ mouse. The representative images are composites from the same western blot image


As mice were being aged for longitudinal behavior studies, a dramatic decrease in survival of *iHip14* ^*Δ/Δ*^ mice, beginning at about 16 weeks of age or 10 weeks post-*Hip14* deletion, was observed (Fig. 7a). Typically, all mice appeared healthy prior to sudden death. Six mice were found with hind limb paralysis prior to being euthanized for other purposes. Post-mortem examination of 14 *iHip14* ^*Δ/Δ*^ mice revealed signs of paralysis in 13, including splayed hind limbs and clenched front paws. One *iHip14* ^*Δ/Δ*^ mouse found with hind limb paralysis was monitored by video (Additional file 1). The paralysis progressed rapidly over 5 h beginning with the hind limbs. Initially, the mouse did not appear distressed and was able to move around the cage and eat. Paralysis progressed until the mouse could no longer move and it was euthanized. A second *iHip14* ^*Δ/Δ*^ mouse was found almost completely paralyzed and was video monitored for a few minutes until it went into respiratory arrest and died (Additional file 2). These data indicate that *iHip14* ^*Δ/Δ*^ mice have dramatically reduced survival due to rapidly progressing paralysis and sudden death.

Additional file 1 Rapidly progressing paralysis in an *iHip14* ^*Δ/Δ*^ mouse over the course of 5 h. An *iHip14* ^*Δ/Δ*^ mouse found with hind limb paralysis was monitored by video. The paralysis progressed rapidly over 5 h, beginning with the hind limbs, from where the mouse was able to move around the cage and eat until it could no longer move and was euthanized. The video lasts a total of 7 min, with the first minute from the beginning of the 5 h and the rest of the video just before the mouse was euthanized. (MOV 12680 kb)



Additional file 2 Sudden death due to rapidly progressive paralysis in an *iHip14* ^*Δ/Δ*^ mouse. A second *iHip14* ^*Δ/Δ*^ mouse was found almost completely paralyzed and was video monitored for a few minutes until it went into respiratory arrest and died. (MOV 6931 kb)


Two *iHip14* ^*Δ/Δ*^ mice survived past 20 weeks of age (10%). One of these mice reached a humane endpoint due to wasting at 43 weeks of age and was euthanized. At the time of euthanasia, it weighed 30% less than its control littermates. The brain was harvested for biochemistry to assess HIP14 protein levels to ensure complete loss of HIP14. Indeed, negligible HIP14 protein was detected, indicating that efficient recombination occurred in this mouse (Fig. 7b).

## Discussion

The most striking phenotype of the *iHip14* ^*∆/∆*^ mice is the rapidly progressing hind limb paralysis leading to sudden death. This was highly unexpected, as there is no survival deficit of *Hip14* ^*gt/gt*^ mice. HIP14 is the most highly conserved PAT, with 99% protein sequence identity between human and mouse and 88% between human and zebrafish as well as 100% conservation of the DHHC active site domain from human to chicken [2]. This high sequence conservation suggests an essential function for the protein, which is supported by the phenotype of *iHip14* ^*∆/∆*^ mice. The constitutive *Hip14*-deficient *Hip14* ^*gt/gt*^ mouse has HD-like neurological deficits [12, 20, 25]; thus, *iHip14* ^*∆/∆*^ mice were expected to develop a similar phenotype. However, the severe phenotype of *iHip14* ^*∆/∆*^ mice shows that HIP14 is crucial for the life of the adult mouse. *Hip14* ^*gt/gt*^ mice develop early onset neurological disease, and these mice express 10% of the endogenous levels of HIP14 in all cells [16, 17], whereas complete loss in >90% of cells in *iHip14* ^*∆/∆*^ adult mice causes a severe phenotype, including sudden death. Thus, complete loss of HIP14 is likely not compatible with survival. It will be interesting to see what happens if HIP14 is fully deleted from conception. However, there may also be developmental compensation that occurs when HIP14 is deleted from conception, likely by other PATs, which cannot occur when HIP14 is deleted in the adult animal.

The *iHip14* ^*∆/∆*^ mice have motor coordination deficits similar to those of *Hip14* ^*gt/gt*^ mice. The motor deficits are dramatic, particularly in the spontaneous climbing test. Also, similar to *Hip14* ^*gt/gt*^ mice [12, 25], *iHip14* ^*∆/∆*^ mice have sensorimotor gating deficits. The PPI test assesses the ability to inhibit an unwanted motor response to a stimulus and is believed to be mediated by the striatum [23]. Both motor dysfunction and PPI impairment suggest striatal dysfunction in the *iHip14* ^*∆/∆*^ mice.

The psychiatric phenotype of the *iHip14* ^*∆/∆*^ mice also suggests striatal dysfunction. The *iHip14* ^*∆/∆*^ mice are hyperactive and reactive to handling, which is consistent with the increased escape response observed during tests of anxiety and depression. This was confirmed when *iHip14* ^*∆/∆*^ mice performed similarly to control mice in the modified light-dark box test that eliminated any avenues for escape. However, anxiety and escape response are likely associated. Thus, it is possible that they become anxious when they are unable to escape a novel environment [36]. Overall, the increased escape response phenotype of *iHip14* ^*∆/∆*^ mice agrees with rodent striatal lesion models with enhanced escape response behavior, providing further evidence of an essential role for HIP14 in striatal function [37].

Interestingly, *iHip14* ^*∆/∆*^ mice have increased forebrain weight due to microgliosis and astrogliosis in the cortex. This may be a downstream response to neuron or circuit dysfunction or may suggest a novel role for HIP14 in glial cell function. Also, although there is clear striatal dysfunction in *iHip14* ^*∆/∆*^ mice, there was no change in striatal volume, unlike the striatal atrophy observed in *Hip14* ^*gt/gt*^ mice [12, 38]. One possible explanation for this discrepancy is that *iHip14* ^*∆/∆*^ mice die before sufficient striatal neuron death for detection by stereology has occurred. There was, however, a decrease in corpus callosum volume, suggesting decreased white matter and potentially axonal degeneration or demyelination.

Further evidence for striatal dysfunction was apparent in the physiology of MSNs. Although there is no change in membrane excitability or surface area in *iHip14* ^*Δ/Δ*^ MSNs, they did display aberrant synaptic transmission. The increase in sEPSC amplitude, decrease in sEPSC frequency, and increased PPR suggest a lower probability of transmitter release but more AMPARs at excitatory synapses in the striatum and/or more glutamate released per synaptic vesicle to the same number of AMPARs. Loss of palmitoylation at either palmitoylation site of GluA1 or GluA2 AMPAR subunits would increase their synaptic expression, which could contribute to these phenotypes [9].

HIP14 has been shown to be a “hub” protein with many interacting partners, and it shares many interactors (not specifically substrates) with HTT, also a “hub” protein with many interactors [39]. In addition to being a PAT, HIP14 has also been shown to have other, non-PAT-related, functions in MAP kinase signaling and magnesium/manganese transport [40–42]. Thus, the phenotype of these mice may be due to loss of palmitoylation of one or multiple crucial proteins, may result from loss of one of these other functions of HIP14, or may be caused by a combination of all these factors.

## Conclusions

This is the first study, to our knowledge, to examine the “conditional knockout” of a DHHC PAT and conclusively demonstrates that HIP14 is essential for life and neuronal integrity. The *iHip14* ^*∆/∆*^ mice have a severe phenotype, different than that of the *Hip14* ^*gt/gt*^ mice, that results in sudden death, striatal dysfunction, and significant astrogliosis and microgliosis. These data highlight the importance of this PAT to neurological function and suggest that palmitoylation is an essential protein modification.

## Methods

### Generation of inducible *Hip14* knockout mice

Xenogen Biosciences (now Taconic Biosciences, Rensselaer, NY, USA) generated the *Hip14* “floxed” mice (*Hip14* ^*F*^) on the FVB/N background strain using a gene targeting strategy where exon 2 was selected as the conditional deletion region, as deletion of this region leads to a frameshift mutation and multiple premature stop codons (Fig. 1a–d). The 5’ and 3’ homology arms and the conditional knockout region (cKO) were amplified from bacterial artificial chromosome DNA and inserted into the targeting vector at the indicated restriction enzyme sites such that the cKO region was flanked by loxP sites (Fig. 1a). A positive selection neo cassette was included and flanked by flippase (Flp) recognition target (FRT) sites, and a negative selection cassette diphtheria toxin A (DTA) was also included to select against random insertion (Fig. 1a). Male FVB/N embryonic stem cells were electroporated with the targeting vector and selected using G418 (Geneticin) resistance and screened for homologous recombination at the 5′ and 3′ homology arms with the WT allele (Fig. 1b) by restriction enzyme digest, southern blot, and PCR. The neo cassette was then removed in positive clones by electroporation with Flp recombinase to mediate recombination between the FRT sites and generate the recombined *Hip14* ^*F*^ allele (Fig. 1c). Neo cassette deletion was confirmed by G418 sensitivity and PCR. *Hip14* ^*F*^ embryonic stem cells were then injected into C57BL/6 J blastocysts to generate male chimeras that were bred with FVB/N females. Resulting white coat progeny indicated germline transmission, and those mice were genotyped using the following primers: the forward primer in the 5′ homology arm in intron 1 (5′-GGAGAATGGTTAGGAAAAGCTCGTACC-3′) and the reverse primer in the cKO region in intron 1 upstream of the first loxP site (5′-GAGGAAAGCATGCAAGAGCACTTCTC-3′).

*Hip14* ^*F/F*^ mice were then crossed to mice expressing Cre-ER^T2^ under the human ubiquitin ligase C promoter, a promoter that will result in ubiquitous Cre expression in all cell types [18] (The Jackson Laboratory, Bar Harbor, ME, USA). The Cre-ER^T2^ transgene expresses Cre recombinase fused to a mutated form of the estrogen receptor that is not activated by estrogen but is activated by the estrogen analog tamoxifen (TM) [18]. This generated mice in which *Hip14* can be deleted at any time point (*Hip14* ^*F/F*^;Cre-ER^T2^). The primers used to genotype at the Cre-ER^T2^ transgene were 5′-GCGGTCTGGCAGTAAAAACTATC-3′ and 5′- GTGAAACAGCATTGCTGTCACTT-3′. Gene deletion was induced using a 5-day TM treatment paradigm by giving a single intraperitoneal injection once a day for 5 days at a dose of 0.2 mg TM/g body weight in 98% corn oil with 2% ethanol (*iHip14* ^*F/F*^ and *iHip14* ^*Δ/Δ*^) or vehicle alone (WT VEH) as previously described [18]. Mice were treated with TM at 6 weeks of age.

### Quantitative real-time PCR

Total RNA was isolated from –80 °C frozen tissues using the RNeasy mini kit (Qiagen, Venlo, The Netherlands). RNA was treated with DNAse I (Life Technologies, Carlsbad, CA, USA) to remove residual genomic DNA. cDNA was prepared from 1 μg total RNA using the SuperScript® III First-Strand Synthesis System (Life Technologies, Carlsbad, CA, USA). Quantitative RT-PCR (qPCR) on the mouse *Hip14* gene using primers spanning exons 1 and 2 (5′-ACCCGGAGGAAATCAAACCACAGA-3′ and 5′-TACATCGTAACCCGCTTCCACCAA-3′) was performed using Power SYBR Green PCR Master Mix (Applied Biosystems, Life Technologies, Carlsbad, CA, USA) and the ABI 7500 Fast Real-Time PCR System (Applied Biosystems, Life Technologies, Carlsbad, CA, USA) under default conditions. Each sample was run in triplicate. Expression levels for mRNA were normalized to β-actin.

### Antibodies

The primary antibodies used were HIP14 polyclonal antibody (in house, 1:400 for immunoblotting), β-tubulin monoclonal antibody (T8328, Sigma, RRID:AB_1844090, 1:5000 for immunoblotting), and NeuN antibody (MAB377, Millipore, RRID:AB_2298772, 1:1000). Biotinylated anti-mouse antibody (BA-9200, RRID:AB_2336171, Vector Laboratories, 1:1000 for immunohistochemistry) was used as a secondary antibody for immunohistochemistry. Fluorescently conjugated secondary antibodies used for immunoblotting were Alexa Fluor 680 goat anti-rabbit (A21076, Molecular Probes, RRID:AB_2535736, 1:10000) and IRDye 800CW goat anti-mouse (610-131-121, Rockland, RRID:AB_220123, 1: 2500).

### Tissue lysis and western blotting analysis

Tissues were homogenized on ice for 5 min in one volume 1% SDS TEEN (TEEN: 50 mM Tris pH 7.5, 1 mM EDTA, 1 mM EGTA, 150 mM NaCl, and 1× complete protease inhibitor cocktail [Roche]) and subsequently diluted in four volumes 1% TritonX-100 TEEN for 5 min for further homogenization. Samples were sonicated once at 20% power for 5 s to shear DNA, and the insoluble material was removed by centrifugation at 14,000 rpm for 15 min.

Proteins in cell lysates were heated at 70 °C in 1× NuPAGE LDS sample buffer (Invitrogen) with 10 mM dithiothreitol (DTT) before separation by SDS-PAGE. After transfer of the proteins onto a nitrocellulose membrane, immunoblots were blocked in 5% milk TBS (TBS: 50 mM Tris pH 7.5, 150 mM NaCl). Primary antibody dilutions of HIP14 polyclonal antibody and β-tubulin monoclonal antibody in 5% BSA PBST (bovine serum albumin, phosphate buffered saline with 5% Tween-20) were applied to the immunoblots at 4 °C overnight. Corresponding secondary antibodies were applied in 5% BSA PBST for an hour. Fluorescence was scanned and quantified with an Odyssey Infrared Imaging system (Li-COR Biosciences, Lincoln, NE, USA) and quantified using the Li-COR software. All error bars represent standard error of the mean.

### Behavior

All behavioral testing was performed with the tester blind to genotype. All testing was performed at 3 months of age (7 weeks post-TM injection). All of the apparatuses were cleaned between mice with 70% ethanol.

#### Spontaneous activity

Spontaneous activity in the dark was measured using the Med Associates activity monitoring system (Med Associates Inc., St Albans, VT, USA) as previously described [20]. Briefly, no later than 1 h after the beginning of the dark cycle following 1 h of acclimatization to the room, mice were placed in the center of the testing chamber (27 × 27 × 20.3 cm) and allowed to freely move about and explore for half an hour. A number of automated readouts were recorded. Ambulatory time is the total time the mouse spent moving while making consecutive beam breaks and ambulatory episodes are the number of times the mouse began ambulating from a resting position.

#### Rotarod and climbing

Fixed-speed and accelerating rotarods were used to assess motor coordination (Ugo Basile, Comerio, Italy) as previously described [20]. Briefly, mice were trained once a day for 3 days on the fixed-speed 18-rpm rotarod for 120 s, and the number of falls and latency to the first fall were recorded. On the fourth day, mice were tested on an accelerating rotarod that accelerates from 5 to 40 rpm over 300 s, and the latency to fall was recorded. The average of three trials is reported.

Motor coordination was also tested on the climbing apparatus as previously described [21]. Briefly, mice were placed inside a closed-top wire mesh cylinder (10 × 15 cm) on the tabletop and were allowed to freely explore for 300 s while being video recorded. A climbing event was recorded when all four paws were off the surface of the tabletop and a rearing event was recorded when the forepaws were off the surface of the tabletop. The climbing time was recorded as the total time from when the fourth paw left the tabletop to when the first paw touched back down.

#### Pre-pulse inhibition (PPI)

PPI was performed using the Startle Response System (San Diego Instruments, San Diego, CA, USA) as previously described [25]. Briefly, mice were placed in a startle chamber and allowed to acclimatize with background noise for 5 min. Mice were then exposed to six trials of a 40-ms, 120-dB startle pulse to test the acoustic startle response. Mice were then exposed to eight blocks of six (48 trials in total) pseudorandomized trials of (1) no stimulus, (2) the 40-ms, 120-dB startle pulse alone, or (3–6) the 40-ms, 120-dB startle pulse preceded 100 ms by a 20-ms pre-pulse of 2, 4, 8, or 16 dB above background. An extra 40-ms, 120-dB pulse was given in four of the eight blocks. Finally, the mice were exposed to another six trials of the 40-ms, 120-dB startle pulse. The inter-trial interval was between 8 and 23 s and was pseudorandomized between trials. PPI is the percentage of decrease in the startle response when a pre-pulse is given prior to the startle pulse and was calculated as the average of six trials per pre-pulse as follows: PPI = [(startle pulse-alone startle) - (pre-pulse + startle pulse startle)]/pulse-alone startle.

#### Porsolt forced swim test

The Porsolt forced swim test was used to assess depressive-like behavior and was performed as previously described [26–28, 43]. Briefly, mice were placed in individual cylinders (25 cm tall × 19 cm wide) filled with room temperature water to a depth of 15 cm and allowed to swim for 6 min while being recorded by video camera. The time spent immobile (not swimming) during the final 5 min was scored.

#### Open field

Open field exploration was used as a test of anxiety-like behaviors as previously described [21]. Briefly, mice were placed into the lower left corner of a 50 × 50 cm open gray Plexiglas box with 16-cm sides in a brightly lit room. The mice were allowed to explore the box for 10 min while being recorded via ceiling-mounted video camera. Videos were live scored using Ethovision XT 7 animal tracking software (Noldus Information Technology), and the total distance traveled and the total time spent in the center of the field were scored as measures of exploratory activity and anxiety-like behavior, respectively.

#### Elevated plus maze

EPM exploration was used as a test of anxiety-like behavior as previously described [31]. Briefly, mice were placed in the center of an EPM 50 cm off the ground with 30 × 10 cm arms, two of which are enclosed by 20-cm walls. Mice were allowed to freely explore the maze for 5 min while a ceiling-mounted camera recorded their activity and Ethovision XT 7 live scored the videos. Distance traveled was used to assess exploratory activity. The time spent in the open arms (open arm duration) and head dips off the edges of the open arms were used as measures of anxiety-like behavior.

#### Light-dark box

The light-dark box was used to test for anxiety in an environment where escape was not possible, i.e., a completely enclosed environment. The Gemini Avoidance System (San Diego Instruments) was used for this purpose; no cues or shocks were used. The door between the two chambers was kept open so mice could freely explore both sides of the box, and on one side a light was shone through the transparent door to create a brightly lit light box. The door on the other side was blacked out to create a dark box. Mice were allowed to freely explore the apparatus, and their activity was recorded using a video camera through the light box side. The total time spent in the light box was scored as a measure of anxiety-like behavior.

#### Sucrose preference

Sucrose preference was used to test for anhedonia, or the loss of pleasure-seeking behaviors, a symptom of depression, as previously described [26, 44]. Briefly, mice were single housed in a full-size cage and were given *ad libitum* access to food and to two water bottles. Mice were allowed to acclimatize to the bottles for 1 week. On day 7 the water in one of the bottles was replaced with a 2% sucrose solution and the mice and both bottles were weighed. Twenty-four hours later the bottles were weighed again and the total fluid and sucrose intake were calculated as g/kg of body weight. Sucrose preference was calculated as follows: sucrose preference = (sucrose intake/total fluid intake) × 100.

#### Neuropathology

All neuropathological studies were conducted as previously described [12, 31]. Mice were anesthetized by intraperitoneal injection of 2.5% avertin and intracardially perfused with ice-cold 4% paraformaldehyde. Brains were harvested and post-fixed in 4% paraformaldehyde for 24 h at 4 °C, and then cryopreserved in 30% sucrose in phosphate-buffered saline (PBS). To determine the brain weight, the olfactory bulbs, paraflocculi, and brain stem were removed prior to weighing. The cerebellum was then removed and weighed separately. Forebrain weight was calculated as brain weight minus cerebellum weight. The forebrain was then flash frozen on dry ice, mounted with Tissue-TEK O.C.T. compound (Sakura Finetek, Torrance, CA, USA), and sectioned coronally on a cryostat (Microm HM 500 M) into 25-μm free-floating sections. Sections were stored until immunohistochemical processing in PBS with 0.01% sodium azide.

A series of 25-μm sections spaced 200 μm apart spanning the striatum were processed for stereological volumetric assessments by staining with NeuN antibody (1:1000, Millipore MAB377) overnight at room temperature to stain all neuronal nuclei. Sections were then stained with biotinylated anti-mouse antibody for 2 h (1:1000, Vector Laboratories BA-9200) and the signal was amplified using the Vectastain ABC kit for 30 min (1:1000, Vector Laboratories) and then detected with 3,3′-diaminobenzidine (DAB, Thermo Scientific). StereoInvestigator software (Microbrightfield Bioscience, Williston, VT, USA) was used to determine striatal, cortical, and corpus callosum volumes by tracing the perimeter of the desired structures; the volumes were determined using the Cavalieri principle.

Two additional series of sections described above were used for glial fibrillary acidic protein (GFAP) and ionized calcium-binding adapter molecule 1 (IBA1) immunohistology. Sections were blocked with 3% H_2_O_2_ in PBS for 30 min, then washed with PBS. Sections were then blocked with 5% normal goat serum (NGS) in PBS-Triton for 30 min. Sections were incubated overnight at room temperature in either monoclonal mouse anti-GFAP-Cy3 antibody (1:500, Sigma-Aldrich) or polyclonal rabbit anti-IBA1 antibody (1:500, Wako), both solutions made in 1% NGS and PBS-Triton. Sections were then incubated for 2 h at room temperature in either biotinylated goat anti-mouse IgG antibody (1:500, Vector) or biotinylated goat anti-rabbit IgG antibody (1:500, Vector), both solutions made in 1% NGS and PBS-Triton. Lastly, sections were incubated with the Vectastain ABC kit (1:1000, Vector) for 30 min and developed with DAB (1:10, Sigma-Aldrich) for 2 min. Sections were mounted on Superfrost Plus microscope slides (Fisher) prior to analysis. Sections were imaged on a Zeiss Axioplan 2 imaging system with a 5× Zeiss Plan-Neofluar objective using a Photometrics Cool Snap HQ camera. Analyses were done using MetaMorph software version 6.3 (Universal Imaging Corporation, Bedford Hills, NY). After delineating the cortex for each image, labeling of GFAP and IBA1 was identified using the threshold held at a constant level with background excluded for all images and then analyzed using the “integrated morphometry” feature. Relative levels of GFAP and IBA1 staining were calculated as the sum of the integrated optical density (IOD) for each image divided by the area of the region selected, then multiplied by the sampling interval (8) and section thickness (25 μm). No staining was observed in a negative control without primary antibody [31].

### Electrophysiology

Mice were transferred to the University of British Columbia (UBC) Animal Research Unit approximately 4–5 weeks following TM injections and all electrophysiological experiments were performed on mice that were approximately 3 months old. Electrophysiological analyses were performed as previously described [14]. Briefly, mice were anesthetized with isoflurane and brains were quickly removed and immediately placed in an ice-cold cutting solution that contained (in millimoles): 125 NaCl, 2.5 KCl, 25 NaHCO_3_, 1.25 NaH_2_PO_4_, 2.5 MgCl_2_, 0.5 CaCl_2_, and 10 glucose. Coronal blocks containing the striatum were then cut on a vibratome (Leica VT1200S) at 400 μm. Striatal sections were transferred to artificial cerebrospinal fluid (ACSF), which was the same as the cutting solution except that it contained 1 mM MgCl_2_ and 2 mM CaCl_2_, and were heated to approximately 32 °C for 30–45 min.

Following recovery, slices were transferred to a recording chamber with ACSF perfused at a rate of 2–3 ml/min. In striatal sections, spiny projection neurons in the dorsal striatum were targeted for recording [14]. For excitatory post-synaptic current (EPSC) recordings in the striatum, picrotoxin (50 μM) was added to the ACSF, but tetrodotoxin (TTX, 0.5 mM) was omitted, as we have previously shown that most EPSCs in our coronal slice preparation are action potential-independent [45]. However, while largely action potential-independent, these are referred to as spontaneous EPSCs (sEPSCs) in the manuscript to indicate the lack of TTX. Glass pipettes (3–6 MΩ) were filled with a potassium gluconate (KGlu) internal solution for sEPSC recordings [46]. The liquid junction potential (theoretical = –15.6 mV) was left uncorrected. sEPSCs were filtered at 1 kHz and digitized at 20 kHz. Where applicable, glutamate release was evoked by an ACSF-filled glass pipette (1 MΩ) placed 200–250 μm dorsal to the recorded cell. Paired pulse ratios (PPRs) were obtained at a –70 mV holding voltage, and various inter-pulse intervals were applied with a stimulus intensity known to generate a response approximately 30–40% of the maximal response. Basic membrane properties were obtained within 60 s following break-in by monitoring the current response to a 10-mV voltage step applied in the membrane test feature in Clampex 10 (Molecular Devices, Sunnyvale, CA, USA). All electrophysiological recordings were acquired and analyzed using the pClamp 10 software bundle.

### Statistics

Data were analyzed using the Student’s *t* test, one-way ANOVA, or two-way ANOVA as indicated using Prism 5 software where all post hoc tests in ANOVA analyses used Bonferroni’s multiple comparison test. Error bars indicate standard error of the mean and in all graphs the mean is indicated. * *p* < 0.05, ** *p* < 0.01, *** *p* < 0.001. In all cases, except for Fig. 6, the number of replicates (*N*) refers to the number of individual mice used and is considered to mean biological replicates. In Fig. 6, *N* refers to the number of cells analyzed from a total of 4 mice per genotype. ANOVA values and exact numbers are listed in Additional file 3.

## Additional files

Additional file 3: One-way and two-way ANOVA values and replicates. (XLSX 40 kb)
Additional file 4 Individual data values for experiments where *N* < 6. (XLSX 44 kb)

## Abbreviations

(DNAJC5): DnaJ heat shock protein 40 homolog
ACSF: Artificial cerebrospinal fluid
AMPAR: α-amino-3-hydroxy-5-methyl-4-isoxazolepropionic acid receptor
ANOVA: Analysis of variance
cKO: Conditional knockout region of *Hip14*
CSP: Cysteine string protein
DARPP-32: Dopamine- and cAMP-regulated neuronal phosphoprotein (PPP1R1B)
DHHC: Asp-His-His-Cys
DTA: Diphtheria toxin A
EPM: Elevated plus maze
EPSC: Excitatory post-synaptic current
Flp: Flippase recombinase
FRT: Flippase recognition target
FVB/N: FVB/NJ mouse strain, Friend virus B NIH Jackson
G418: Geneticin
GABA_A_: γ-aminobutyric acid A (receptor)
GluA1: AMPA receptor subunit 1 (GRIA1)
GluA2: AMPA receptor subunit 2 (GRIA2)
GluN2A: NMDA receptor subunit 2A (GRIN2A)
GluN2B: NMDA receptor subunit 2B (GRIN2B)
HD: Huntington disease
HIP14: Huntingtin interacting protein 14 (ZDHHC17)
HTT: HD disease-causing protein huntingtin
loxP: Locus of X-over P1, 34 bp Cre recombinase sequence from P1 bacteriophage
MAP: Mitogen-activated protein
MSN: Medium spiny neurons
NMDAR: *N*-methyl-d-aspartate receptor
PAT: Palmitoyl acyltransferase
PPI: Pre-pulse inhibition
PPR: Paired pulse ratio
PSD95: Post-synaptic density protein 95 (DLG4)
SNAP25: Synaptosomal-associated protein 25
STREX BK: Stress-regulated exon splice variant of the calcium- and voltage-activated potassium channel
SYT1: Synaptotagmin 1
TM: Tamoxifen
WT: Wild type.
